# New genomic insights into the conformation of Lipizzan horses

**DOI:** 10.1038/s41598-023-36272-4

**Published:** 2023-06-02

**Authors:** A. I. Gmel, G. Brem, M. Neuditschko

**Affiliations:** 1 Equine Department, Vetsuisse Faculty, University of Zurich, Winterthurerstrasse 260, 8057 Zurich, Switzerland; 2 Animal GenoPhenomics, Agroscope, Rte de La Tioleyre 4, 1725 Posieux, Switzerland; 3 Institute of Animal Breeding and Genetics, Veterinary University Vienna, Veterinärplatz 1, 1220 Vienna, Austria

**Keywords:** Genetics, Animal breeding, Genetic association study, Heritable quantitative trait, Anatomy, Musculoskeletal system

## Abstract

Conformation traits are important selection criteria in equine breeding, as they describe the exterior aspects of the horse (height, joint angles, shape). However, the genetic architecture of conformation is not well understood, as data of these traits mainly consist of subjective evaluation scores. Here, we performed genome-wide association studies on two-dimensional shape data of Lipizzan horses. Based on this data, we identified significant quantitative trait loci (QTL) associated with cresty neck on equine chromosome (ECA)16 within the *MAGI1* gene, and with type, hereby differentiating heavy from light horses on ECA5 within the *POU2F1* gene. Both genes were previously described to affect growth, muscling and fatty deposits in sheep, cattle and pigs. Furthermore, we pin-pointed another suggestive QTL on ECA21, near the *PTGER4* gene, associated with human ankylosing spondylitis, for shape differences in the back and pelvis (roach back vs sway back). Further differences in the shape of the back and abdomen were suggestively associated with the *RYR1* gene, involved in core muscle weakness in humans. Therefore, we demonstrated that horse shape space data enhance the genomic investigations of horse conformation.

## Introduction

Conformation refers to all exterior, visible traits of the overall morphology of an animal (height, segment lengths, joint angles, shape of the head, neck, back or croup). Horses have been selected based on conformation traits for centuries, resulting in diverse breeds with specific use (heavy type draught horses, light type sport horses, small ponies, etc.)^1–3^. Some traits, such as the concave (dished) head shape in the Arabian horse, are breed-specific and essentially aesthetic. Other traits are relevant for health (limb angles) or influence performance (slope of the shoulder or croup)^4^. While some of these traits, such as height length and angle measurements, can be objectively quantified, those describing shapes, proportions or having an aesthetic component are usually scored by experts of a particular breed. Conformation scores have been shown to be subjective and unreliable in many breeds, and are therefore difficult to be included in genetic analyses^5–8^. To date, only one recent genome-wide association analysis (GWAS) has identified a quantitative trait locus (QTL) for the conformation of the croup in the Icelandic horse based on subjective scoring data^9^. Advances in data imagery through the field of geometric morphometrics have led to the development of the horse shape space model, tracing the contour and certain anatomical landmarks on standardized photographs of horses^10^. Shape space data has been shown to be more heritable and reliable than traditional scores for the shoulder joint and fetlock joint angles of the front and hind limb in Franches–Montagnes (FM) horses^11^. Furthermore, a GWAS of joint angles from the horse shape space model applied to FM and Lipizzan horses revealed novel QTL for poll angle and elbow joint angle^12^. To date, two-dimensional (2D) geometric morphometric data have been already used to explore the genetic architecture of morphological differences in mollusks^13^, sorghum^14^, or house flies^15^, while three-dimensional (3D) data has been applied to study facial traits in humans (summarized in^16^), cats^17^ and dogs^18^.

Our aim was to explore the genetic architecture of conformation in horses. For this purpose, we used 2D shape data from the Lipizzan horse breed. The Lipizzan breed is considered a baroque riding horse, traced back to a few known Italian and Spanish founder animals born in the eighteenth century. Between 1776 and 1945, Arabian horses were also introgressed to “ameliorate” its type, affecting the conformation of the head, neck, withers and extremities^19^. Currently, Lipizzan horses of a lighter type are associated with higher Arabian admixture proportions and have a more concave head and more salient withers^20^. Consequently, there is a relatively large phenotypic variation in the Lipizzan horse, which makes it an ideal model to explore conformation differences in the horse in a GWAS, while avoiding population stratification present when comparing different breeds.

Here, we report QTL for shape-derived conformation traits representing a gradient from a heavy to light type, from a roach to sway back, as well as for the shape of neck. These findings may have implications for the selection of Lipizzan horses, and potentially, the domesticated horse in general.

## Results

### Phenotypic shape variation

The first twelve principal components (PCs) accounting for more than 1% of the shape variance (246 landmarks) were visualised in Supplementary Fig. 1. Based on the warp grids along the twelve PCs, eight PCs (PC1, PC2, PC4, PC5, PC8, PC9, PC10 and PC12) were excluded from the downstream analyses, as they were strongly influenced by the posture (head, neck and or limbs) of the horse (e.g. the first PC, accounting for 40.27% of the variance, mainly described the gradient of the head position). Compared to the discarded PCs, the remaining four PCs were associated with shape differences of the horses. The third PC, accounting for 8.68% of the variance, highlighted the width and shape of the neck, with the most extreme shape reminiscent of a “cresty neck”. The sixth PC showed a gradient between a horse with a shorter sway back to a horse with a longer and straight or slightly roach back and explained 4.50% of the variance. The seventh PC, accounting for 3.59% of the variance, described differences in the shape of the Lipizzan horse previously associated with Arabian genetic contributions (lighter horse type)^20^. The eleventh PC, explaining 1.32% of the variance, described a gradient between a horse with a larger neck, torso and a more sloping croup compared to a horse with a slimer torso and horizontal croup.

For the shapes of the body parts (torso and neck), we applied the same principle and selected the second and the fifth PC accounting for 21.38% and 3.51% of the torso and neck shape variance, respectively.

**Figure 1:**
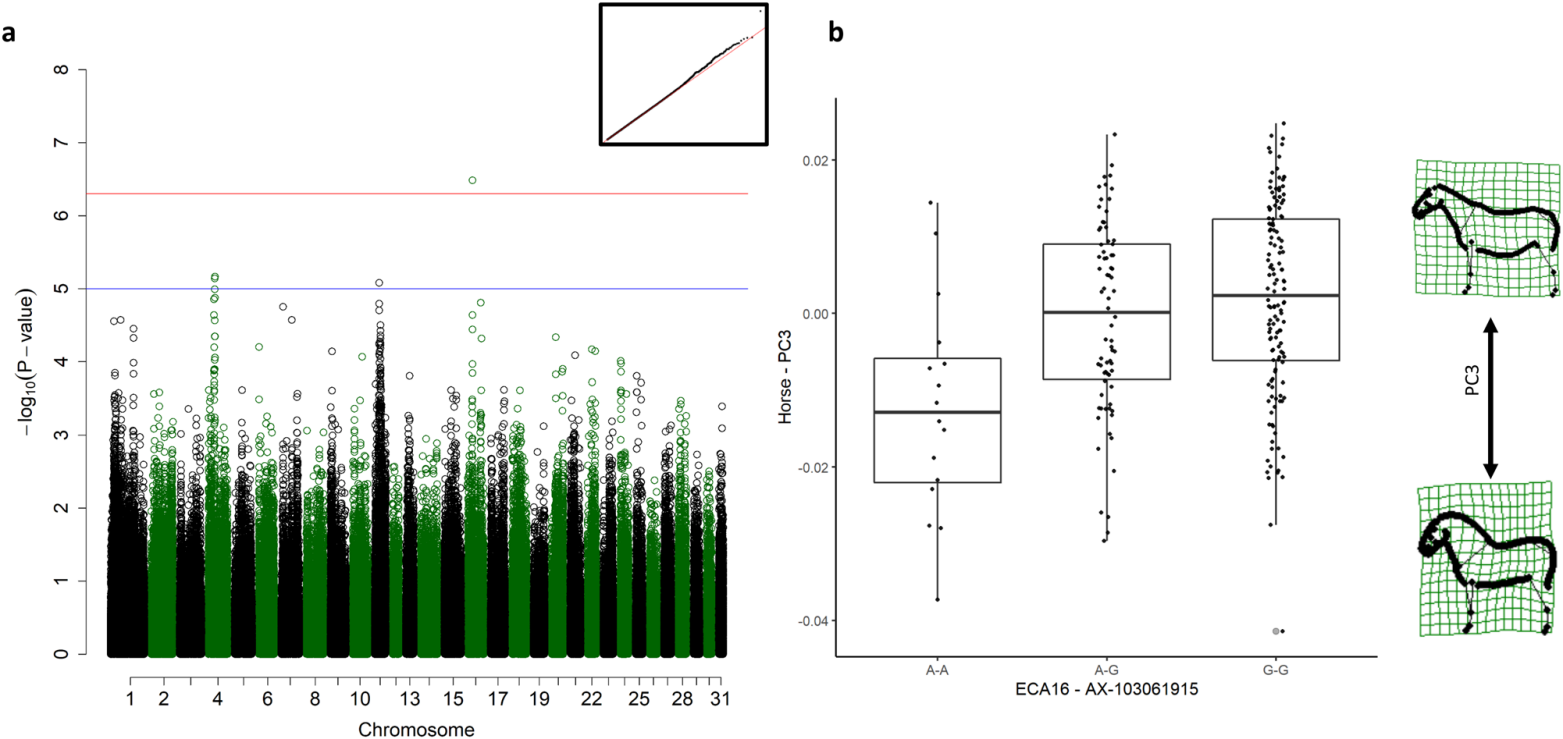
Genome-wide association study (GWAS) for the scores from the third principal component (PC3). (**a**) Manhattan plot with the red line representing the significance threshold corrected for the effectively independent single nucleotide polymorphisms (SNPs) (*p*_ind_ < 4.98 × 10^–7^). The inset on the right-hand corner shows the quantile–quantile (Q–Q) plot with the observed *p*-value plotted against the expected one. (**b**) Boxplots representing the genotype effect of the SNP on chromosome 16 on the shape of the horse. The horizontal line shows the median, the box extends from the lower to the upper quartile, and the whiskers to 1.5 × the interquartile range above the upper quartile or below the lower quartile. On the right-hand side, the extreme shapes for PC3 (shape with the highest PC3 score on top, shape with the lowest PC3 score on the bottom) are visualised in deformation grids.

### Cresty neck

The scores from PC3 were significantly associated with a single SNP on ECA16 at 25,245,630 (*p* = 3.25 × 10^–07^), within the *membrane-associated guanylate kinase, WW and PDZ containing 1* (*MAGI1)* gene (ECA16: 24,927,284–25,524,757, Fig. 1a). Horses with the alternative A-A genotype had a much larger (cresty) neck than heterozygous A-G or homozygous G-G horses (Fig. 1b).

### Spinal curvature

**Figure 2:**
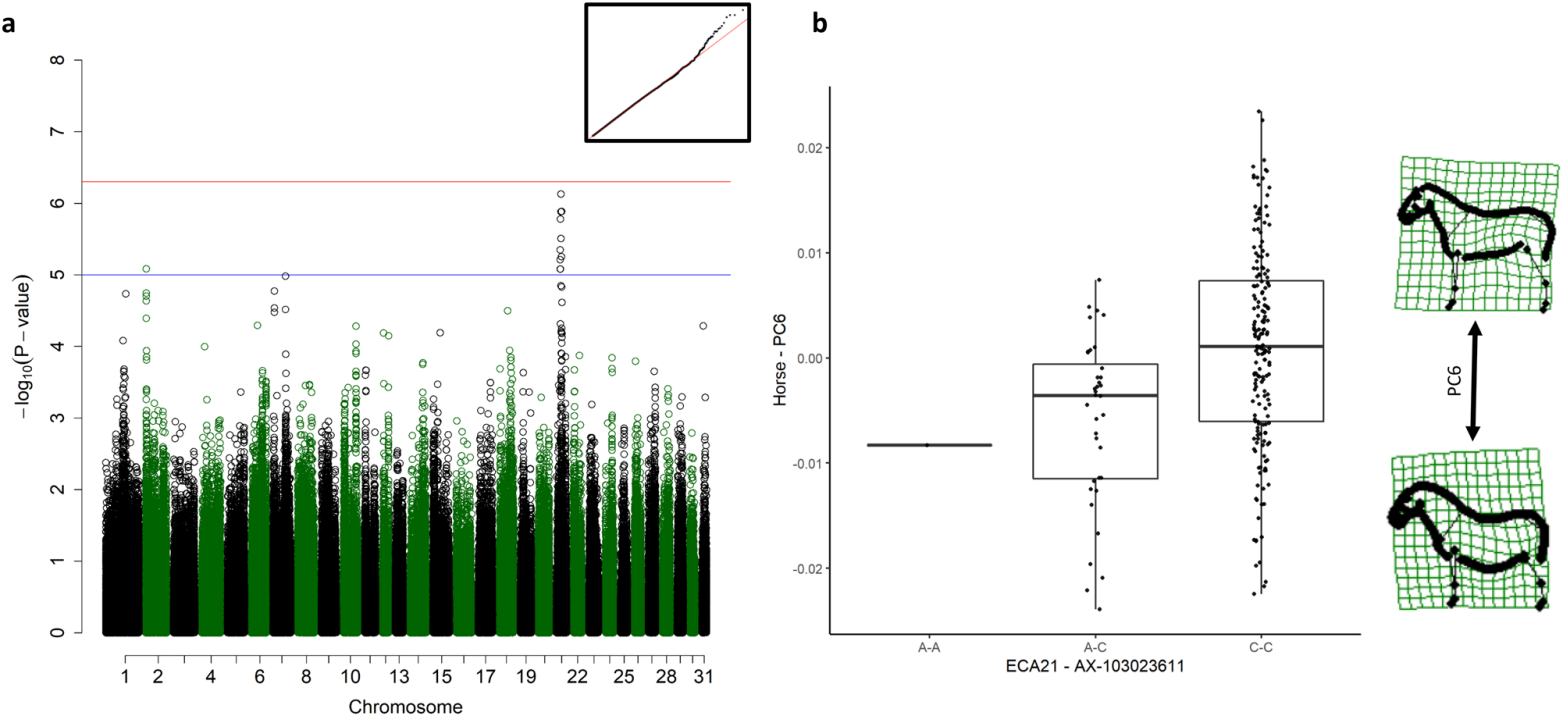
Genome-wide association study (GWAS) for the scores from the sixth principal component (PC6). (**a**) Manhattan plot with the red line representing the significance threshold corrected for the effectively independent single nucleotide polymorphisms (SNPs) (*p*_ind_ < 4.98 × 10^–7^). The inset on the right-hand corner shows the quantile–quantile (Q–Q) plot with the observed *p*-value plotted against the expected one. (**b**) Boxplots representing the genotype effect of the SNP on chromosome 21 on the shape. The horizontal line shows the median, the box extends from the lower to the upper quartile, and the whiskers to 1.5 × the interquartile range above the upper quartile or below the lower quartile. On the right-hand side, the extreme shapes for PC6 (shape of the horse with the highest PC6 score on top, shape of the horse with the lowest PC6 score on the bottom) are visualised in deformation grids.

The scores from PC6 were significantly associated with a QTL on ECA21 spanning 10 SNPs from 22,096,555–30,582,615 (Fig. 2a). The best associated SNP (*p* = 7.42 × 10^–07^) was located at 26,328,965 near the *Prostaglandin E Receptor 4* (*PTGER4)* gene (ECA21: 26,143,605–26,157,288). Horses homozygous for the reference allele (C–C genotypes) tended to have a rounder croup, a hyperkyphotic back (roach back) and a tight abdomen (Fig. 2b), while A-C and A-A genotypes showed a more distended abdomen, swayback and horizontal croup.

### Type

**Figure 3:**
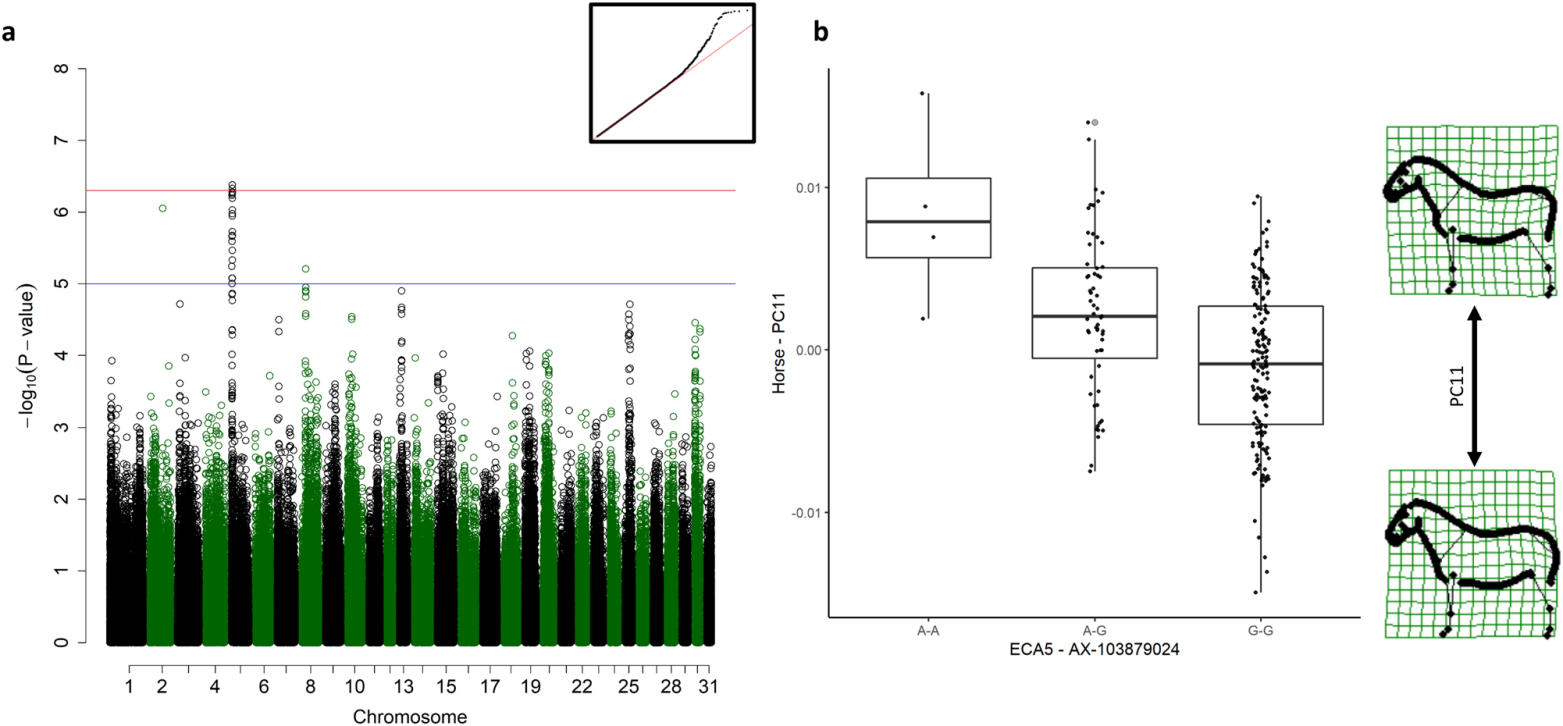
Genome-wide association study (GWAS) for the scores from the eleventh principal component (PC11). (**a**) Manhattan plot with the red line representing the significance threshold corrected for the effectively independent single nucleotide polymorphisms (SNPs) (*p*_ind_ < 4.98 × 10^–7^). The inset on the right-hand corner shows the quantile–quantile (Q–Q) plot with the observed *p*-value plotted against the expected one. (**b**) Boxplots representing the genotype effect of the SNP on ECA5 on the shape. The horizontal line shows the median, the box extends from the lower to the upper quartile, and the whiskers to 1.5 × the interquartile range above the upper quartile or below the lower quartile. On the right-hand side, the extreme shapes for PC11 (shape of the horse with the highest PC11 score on top, shape of the horse with the lowest PC11 score on the bottom) are visualised in deformation grids.

The scores from PC11 were significantly associated with a large QTL on ECA5 spanning 22 SNPs from 1,552,569–4,094,935 (Fig. 3a). The best-associated SNP (*p* = 4.15 × 10^–07^) was located at 3,879,741 in the *POU Class 2 Homeobox 1 (POU2F1,* also known as *OCT1* or *OTF1)* gene (ECA5: 3,876,009–4,050,835). The horses with an A-A genotype (reference allele) were of a lighter type, with less neck and hindquarter musculature and a more horizontal croup, while G-G horses were heavier built, with a sloping, muscular croup (Fig. 3b). The heterozygous horses were of medium size. For the scores from PC7, which was also associated with type, no significant nor suggestive association was determined.

### Withers’ salience

The scores from PC5 of neck landmarks were significantly associated with a single SNP on ECA22 at 20,846,113 (*p* = 2.88 × 10^–07^), near the Transglutaminase 3 (TGM3) gene (ECA22: 20,818,610–20,858,852) (Supplementary Fig. 2a). Heterozygous T-C horses had little withers’ salience and a larger underside of the neck, while homozygous C–C horses had a tendency for a swan neck and clearly defined withers (Supplementary Fig. 2b). There were no homozygous T-T horses in the dataset.

### Abdominal shape

**Figure 4:**
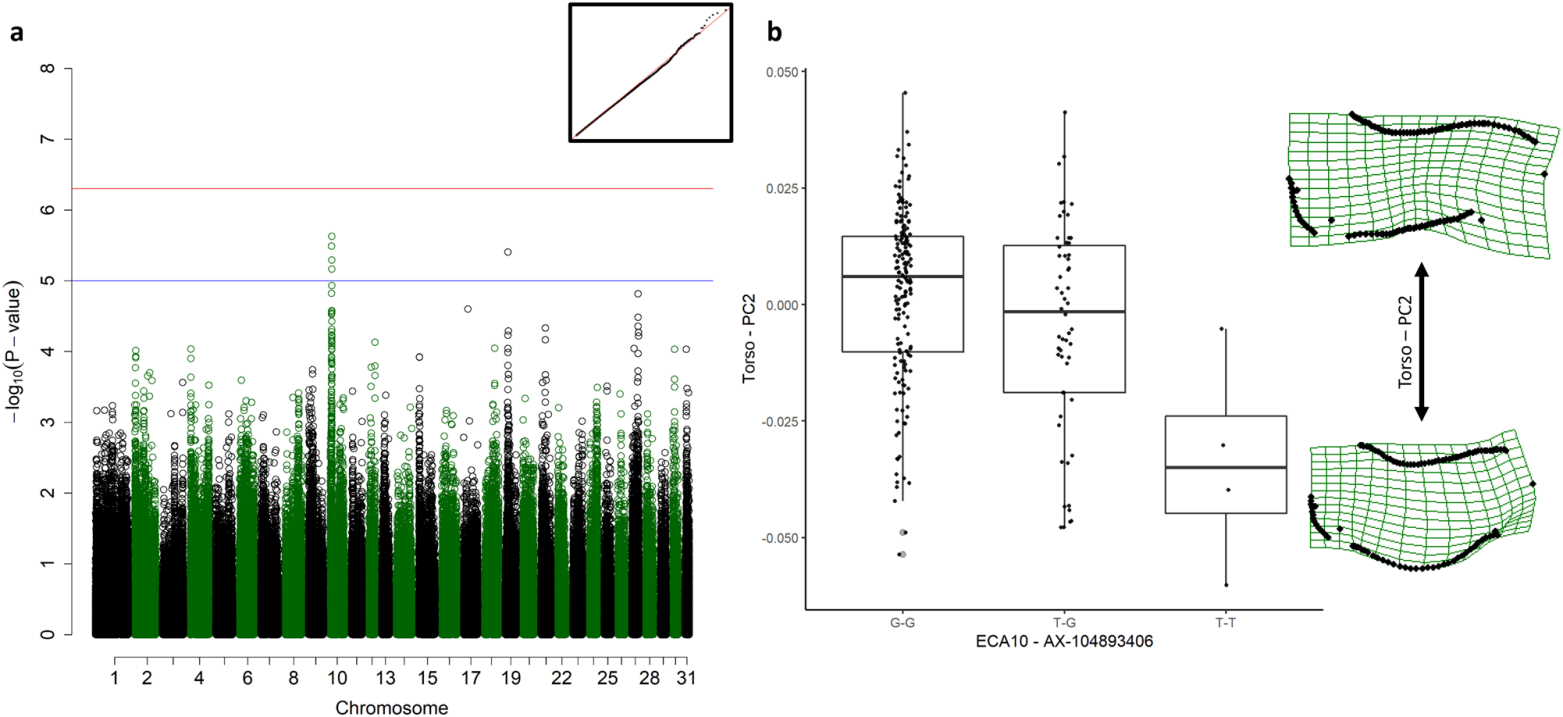
Genome-wide association study (GWAS) for the scores from the second principal component of the torso landmarks (Torso PC2). (**a**) Manhattan plot with the red line representing the significance threshold corrected for the effectively independent single nucleotide polymorphisms (SNPs) (*p*_ind_ < 4.98 × 10^–7^). The inset on the right-hand corner shows the quantile–quantile (Q–Q) plot with the observed *p*-value plotted against the expected one. (**b**) Boxplots representing the genotype effect of the SNP on ECA10 on the shape of the torso. The horizontal line shows the median, the box extends from the lower to the upper quartile, and the whiskers to 1.5 × the interquartile range above the upper quartile or below the lower quartile. On the right-hand side, the extreme shapes for PC2 of the torso (shape of the torso with the highest PC2 score on top, shape of the torso with the lowest PC2 score on the bottom) are visualised in deformation grids.

The scores from PC2 of torso landmarks were suggestively associated with a QTL on ECA10 including four SNPs spanning from 8,390,393 to 9,703,042, with the best-associated SNP (*p* = 2.34 × 10^–06^) at 9,664,363, embedded in the *Ryanodine receptor 1 (RYR1)* gene (ECA10: 20,818,610–20,858,852) (Fig. 4a). Homozygous T-T horses had a sagging abdomen and a tendency towards a sway back (Fig. 4b). G-T and G-G horses had a straighter back and abdominal line.

### Heritability of shape-derived conformation traits

The genome-wide heritability (h^2^_SNP_) of the five shape-derived conformation traits ranged from 0.09 ± 0.12 (PC11) to 0.63 ± 0.14 (PC3, Table 1). The standard errors were therefore fairly high in comparison, especially for traits with a lower h^2^_SNP_ (PC11, PC5 of neck landmarks, PC2 of torso landmarks). The aforementioned QTL, including surrounding SNPs ± 2.5 Mb, explained 33% up to 99% of h^2^_SNP_, while the standard errors were smaller.

**Table 1:** Heritability of phenotypic traits across all SNPs (h^2^_SNP_) and the 5-Mb QTL windows (h^2^_QTL_), including their standard error (SE).

Trait	h^2^_SNP_ ± SE	SNP with lowest *p*-value in GWAS	*P*-value from GWAS	ECA	Position of redefined QTL (EquCab 3.0)	h^2^_QTL_ ± SE	Proportion h2_SNP_ explained by h^2^_QTL_ (%)
PC11	0.09 ± 0.12	AX-103879024	4.15E-07	5	1,379,741–6,379,741	0.09 ± 0.05	99
PC3	0.62 ± 0.14	AX-103061915	3.25E-07	16	22,745,630–27,745,630	0.20 ± 0.10	33
PC6	0.54 ± 0.14	AX-103023611	7.42E-07	21	23,828,965–28,828,965	0.34 ± 0.11	63
PC5 (neck)	0.19 ± 0.14	AX-104245250	2.88E-07	22	18,346,113–23,346,113	0.10 ± 0.07	54
PC2 (torso)	0.21 ± 0.14	AX-104893406	2.34E-06	10	7,164,363–12,164,363	0.12 ± 0.06	59

## Discussion

### Shape variation

The first two PCs, accounting for more than 50% of the shape variation, were consistent with previous results applying the horse shape space model on either Lipizzan^6,10,20,21^ or FM^5,11^ horses. The posture of the head systematically has the greatest effect on shape variation overall and cannot be entirely standardised in the field. Despite explaining most of the shape variance, PC1 (head height) and PC2 (poll flexion–extension) were not as heritable as PC3 describing the width of the neck in the FM horse^11^. Therefore, it is important to notice, that posture effects might account for the majority of shape variance and need to be considered in the selection of PC associated with conformation of the horse.

### Cresty neck

Only a single SNP within the *MAGI1* gene was significantly associated with the shape and width of the neck. This gene has not been described previously in equine studies, while in sheep, *MAGI1* was associated with average daily gain and Kleiber ratio (average daily gain/end weight^0.75^) in 6- to 9-month-old sheep^22^. Furthermore, this gene was reported in a selection signature analysis differentiating two sheep breeds, the Sarda and Sardinian Ancestral Black, the latter having a much smaller body size^23^. In the horse, a cresty neck is the consequence of localised fat deposits in the dorsal neck region delineated by the nuchal ligament^24,25^ and is highly prevalent in Baroque breeds such as Pura Raza Español (PRE, or Andalusian), Lusitano, Morgan or Paso Fino horses^26^. Cresty neck is an undesirable trait in the PRE, and horses with high cresty neck scores are greatly penalised during breeding shows^26^. Furthermore, there are some health concerns associated with cresty neck, as it has been associated with insulin dysregulation, one of the risk factors for equine metabolic syndrome, especially when measuring the neck circumference near the base of the neck^27,28^. Interestingly, *MAGI1* variants have previously been associated with glucose response^29^ and insulin resistance^30,31^ in human studies. We suggest that the identified QTL in the *MAGI1* gene might be associated with cresty neck in Lipizzan horses by affecting insulin regulation. Further studies including additional data from other Baroque breeds including horses with or without cresty neck, in combination with metabolic data such as blood insulin and glucose levels, are required to confirm this QTL.

### Type

The QTL associated with type is located within the *POU2F1* gene, a transcription factor expressed in many different tissues^32^. In the Brazilian Nellore cattle, a QTL containing the *POU2F1* gene explained 41% of genetic variance in conformation and 29% in muscling scores^33^ and was also identified as one of the major transcription factors regulating growth and fatty deposits in Iberian × Landrace pigs^34^. In humans, certain Pou2f1 isoforms were either heavily up- or downregulated by repulsive guidance molecule a (RGMa), important in skeletal cell differentiation, cell fusion and hypertrophy^35^. *POU2F1* variants were also associated with human Type 2 Diabetes Mellitus^36,37^, and it is common that adults affected by Type 2 Diabetes lose skeletal muscle mass^38^. Therefore, the identified QTL is likely associated with both muscle mass and fatty deposits.

### Spinal curvature: skeletal stiffness or muscle weakness?

The scores from PC6, representing the shape variation between a sway and a roach back, were suggestively associated with a QTL near the *PTGER4* gene. In humans, this gene has previously been associated with the severity of ankylosing spondylitis (also known as Morbus Bechterew)^39,40^. Main clinical signs include lower back pain, spinal stiffness and loss of mobility^41,42^. Hyperkyphosis (roach back) is particularly common in young men^43^. The hip and shoulders may also become affected^44,45^, with an observed shift between movements of the shoulder girdle and the pelvis^46^. Although we are comparing phenotypes between bipedal humans and quadrupedal horses, the shape variance explained by PC6 was still consistent with some of the aforementioned symptoms. The higher extreme showed a horse shape with a roach back and sloping croup, as opposed to the lowest extreme of a sway backed horse (hyperlordosis) with a horizontal croup, although we have no information on actual clinical signs in this dataset of horse photographs. Follow-up studies should include radiographs of genotyped horses with extreme roach back or sway back, as well as an objective movement analysis of homozygous horses from both genotype groups to determine whether the conformation would affect the gait quality of the horse, as stride length and over-tracking distance are highly dependent on the movement of the pelvis at walk, and to a lesser extent, at trot^47,48^. Previous genomic research investigated juvenile onset lordosis in the American Saddlebred horse identifying a large haplotype on ECA20 potentially involving the *TRERF1*, *TAF8* and *C6orf132* genes^49^. The causal variant could not be identified yet, which the authors attributed to the phenotyping method and the complexity of the trait^50^. The new QTL on ECA21 may be one of the contributing genetic factors influencing the severity of lordosis. However, this should be confirmed by multi-breed studies to better understand whether this QTL is only present in Lipizzan horses. There have been no genetic studies on roach back in the horse to date, although it is also an undesirable trait in horse breeding, similarly to sway back.

The shape of the torso was suggestively associated with a QTL containing *RYR1*. *Ryanodine receptor 1 gene* mutations have been associated with malignant hyperthermia in many mammals including pigs^51^, dogs^52^ and horses^53^, but also with myopathies such as central core disease (CCD), Multiminicore Disease (MmD), Centronuclear myopathy (CNM) and axial hereditary myopathy^54–56^. Symptoms vary, but commonly include proximal muscle weakness, scoliosis and hip girdle weakness. In the case of the axial hereditary myopathy, pronounced lumbar hyperlordosis is frequent^56^. Joint hypermobility may lead to hip dislocations, although for MmD cases the spine is very rigid^55^. In the horse, the gravitational pull of the viscera will over time extend the back towards lordosis, especially in the case of pregnant mares. This effect can be counteracted by well-developed ventral musculature, which can flex the back^57^. The extreme phenotype of the equine torso indicates a weakness of the abdominal muscles, which may cause the back to sag towards the ground.

For the shape of the back and abdomen, we have discovered two genes with different actions, but similar phenotypes. While *PTGER4* could influence the shape of the spine due to osseous fusion by increasing spinal rigidity, *RYR1* could weaken the abdominal musculature, causing the spine to bend as a consequence. These results underline the complex phenotype and genetic architecture of conformation traits.

### Neck shape and mane

The scores from PC5 of the neck landmarks were significantly associated with a single SNP near the *TGM3* gene. *TGM3* is involved in skin and hair physiology. Mutations in this gene were associated with uncombable hair syndrome in humans^58^ and wavy hair (“wellhaarig” phenotype) in mice^59^. *TGM3* was identified as one of the cis-regulatory elements involved in the adaptation of the Yakutian horse to the Arctic environment, probably by increasing the thickness of the coat^60^. It is unclear how this gene might affect the shape of the neck and withers. It is possible that if the structure of the mane hair is more rigid, the outline of the neck would be overestimated during the digitising process. *TGM3* was also associated with some forms of head and neck cancers, which would suggest a regulatory function in this anatomical region^61^.

### Heritability of shape-derived conformation traits

The h^2^_SNP_ of the five shape-derived morphological traits were low (< 0.10) to high (> 0.40) and exceeded high standard errors, most likely due to the small sample size. Interestingly, the redefined QTL accounted for 33% up to 99% of h^2^_SNP_. The scores from PC3 associated with the neck width variation had the highest h^2^_SNP_. This was consistent with findings from a study of the shape space in FM horses, where the PC describing the neck width also had a high heritability, albeit pedigree-based^11^.

### Current limitations and future implications

This study has revealed several novel QTL associated with conformation in the Lipizzan horse. However, the sample size was small, and the applied SNP data did not include whole-genome sequence information. The horse shape space model is an efficient high-throughput phenotyping method to infer objective information on different aspects of equine conformation (PCs and joint angles^5,11,12^). Based on this method it becomes feasible to derive objective conformation traits of hundreds or thousands of horses independently from breeding judges, which simultaneously improves the data quality and quantity for genetic analyses. Furthermore, shape-derived conformation traits can be pooled across breeds and therefore support, multi-breed comparisons. However, there are currently two major limitations: the posture of the horses when analysing a single photograph, and the association of the PCs with conformation, which remains subjective. Excluding the effect of posture, by standardising the position of the head and limbs during photo taking, or by using the mean of several shapes of the same horse photographed in different postures, should result in PCs associated with differences in conformation.

In conclusion, with the application of the horse shape space model, it became feasible to identify several novel QTL associated with complex conformation traits in the Lipizzan horse. Additional studies including larger sample sizes, whole-genome sequencing and different breeds will improve our understanding of the functional genomics of the reported QTL.

## Material and methods

### Animals

In total, 229 horses from the Lipizzan horse breed were included in the analyses (102 females and 127 males), born between 1987 and 2013; median of 2005). Samples were collected in the studs of Piber (Austria), Đakovo (Croatia), Topol’čianky (Slovakia) and Szilvasvárad (Hungary) between 2014 and 2017, following national rules and regulations. All horses were genotyped on the commercial Axiom™ Equine genotyping Array containing 670,795 evenly distributed single nucleotide polymorphisms (SNP)^62^. After quality control, 226 horses remained for analysis.

### Phenotyping

Each horse was phenotyped based on one unique photograph from the side. The horses were placed in open posture, as previously described^5,10,11^. The photographs were digitised with tpsDig2^63^ following the outline horse model described in Druml et al.^10^: 18 anatomical and 15 somatometric landmarks combined with eight outlines (head upper side, neck, back, hindquarter, belly, chest and neck lower side, jaw and head lower side), for a total of 246 landmarks (Supplementary Fig. 3). After a generalised Procrustes analysis (GPA) which scales, rotates and centres every shape according to the mean configuration of the sample, the 213 semi-landmarks were slid to minimize the amount of bending energy between each configuration and the average of all specimen in an iterative process with the software tpsRelw v1.70^64^. The shape variation in the 246 landmarks was then summarised in principal components (PCs), where each score on a PC corresponded to one specific horse, and therefore to a specific shape pattern. The PCs explaining more than 1% of the variance for the outline horse model were considered for GWAS. From the 246 landmarks of the full horse model, we also extracted landmark configurations for the neck and torso (Supplementary Fig. 3), each summarised in PCs. The PCs were visualised using warp grids, and the main variation of each PC was described.

### Statistics and reproducibility

The reproducibility of the horse shape space model has been extensively studied elsewhere: The digitisation process of single horse photographs was highly repeatable (same image and digitiser) and reproducible (same image, different digitisers)^5,10^. The consistency of the shape (same animal, different images) was influenced by age and posture^5^. Therefore, we corrected for age in our statistical analyses and excluded PCs affected by posture.

### Genome-wide association studies

After removing SNPs not located on the autosomes, with a minor allele frequency below 5%, a genotyping rate below 90% or departing from Hardy–Weinberg equilibrium (HWE) at *p* < 0.0001 using PLINK^65,66^, 361,411 genome-wide SNPs were available for analysis. Genome-wide association studies (GWAS) were performed using a polygenic model (*polygenic_hgml*) in the R-package GenABEL^67^ with the R version 3.4.1^68^, taking into account sex (male or female) and age by categories (three to four, five to eight, nine to sixteen and over sixteen years old) as covariates in addition to the genome-derived kinship matrix. The age categories were previously defined by Druml et al.^21^ for the Lipizzan horse. The significance of each SNP was extracted using *mmscore*.

The results were visualised with Manhattan plots, with a suggestive significance threshold at *p* < 10^–5^, and the significance threshold as the effective number of independent loci (*p*_ind_) by pruning the 361,411 SNPs for linkage disequilibrium (LD). Using a 50-kb sliding window size, a 5-kb window step size and an r^2^ exclusion threshold of 0.5, resulting in an LD-independent *p*-value threshold of *p*_ind_ < 4.98 × 10^–7^. We also evaluated the quantile–quantile (Q–Q) plots for the inflation of small p-values, hinting at false positive association signals. The effect of the SNP on the phenotype was evaluated graphically with boxplots to visualize differences in phenotypic values. We investigated which genes were located near significant SNPs using the NCBI Genome Data Viewer, based on the EquCab 3.0 reference genome assembly^69^. Genes functions in horses, humans and other livestock species were investigated in current published peer-reviewed literature (last research: 03.05.2022).

### Heritability estimates of shape-derived conformation traits

For each reported QTL, we estimated the genome-wide heritability (h^2^_SNP_) using the GCTA software^70^ by including all available SNPs simultaneously into a genetic relationship matrix (grm), followed by a restricted maximum likelihood estimation (REML) with the grm including the aforementioned covariates age categories and sex as predictors and each conformation trait as the response variable^71^. To estimate the proportion of the heritability explained by the SNP with the highest-log10 *p*-value, we defined a QTL window of ± 2.5 Mb surrounding the most significant SNP in order to establish an informative grm^72^. Thereafter, we estimated the heritability of the 5-Mb QTL (h^2^_QTL_) with the covariates age categories and sex, and calculated the proportion of the heritability explained by the 5-Mb QTL compared to all genome-wide SNPs (h^2^_QTL_/h^2^_SNP_).

### Ethical approval

This study was discussed and approved by the institutional Commission for Ethics and Animal Welfare, University of Veterinary Medicine, Vienna, protocol number: ETK-06/05/2015, in accordance with GSP guidelines, national legislation and ARRIVE guidelines.

## Supplementary Information

Supplementary Figures.

## Data Availability

The data that support the findings of this study are available from project consortium FFG project number 843464; Veterinary University Vienna, Xenogenetik and five European state stud farms, but restrictions apply to the availability of these data. Data are available from the corresponding author upon reasonable request and with permission of project consortium, FFG project number 843464; Veterinary University Vienna, Xenogenetik and five European state stud farms.
