# Supplementary material for: New genomic insights into the conformation of Lipizzan horses
Source: Sci Rep. 2023 Jun 2;13:8990. doi: 10.1038/s41598-023-36272-4 (PMC10238546; doi:10.1038/s41598-023-36272-4)

Supplementary Figure 1 : Warp grid representation of the extreme shapes describing all relative warp axes (PCs) explaining > 1% of the variance with their textual interpretation into conformation traits

a) PC1 - Head position (up or down)

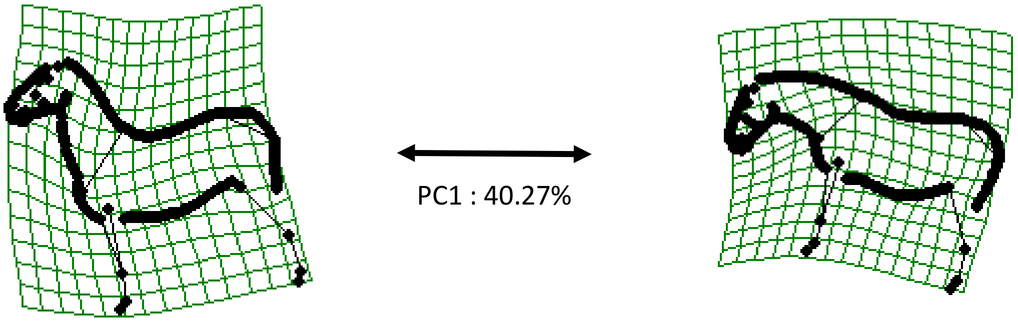

b) PC2 – extension-flexion at the poll

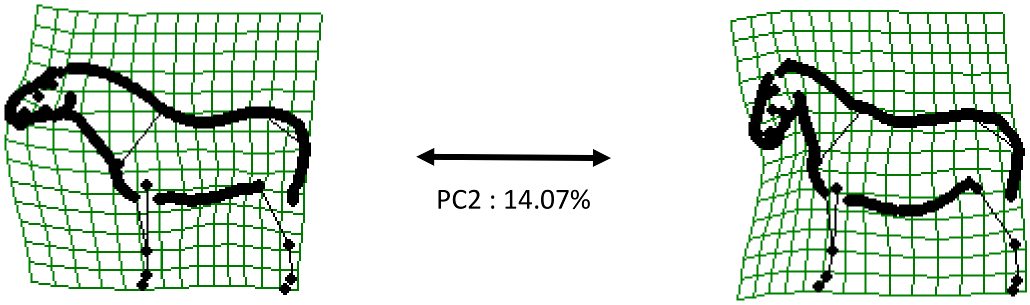

c) PC3 - Neck width (cresty neck)

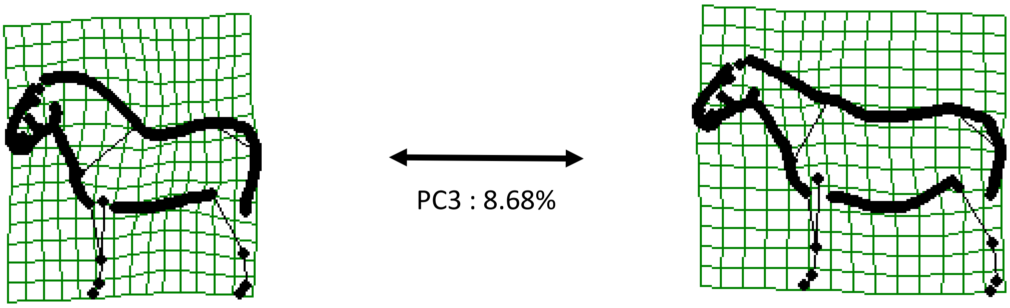

d) PC4 - Hind limb camped out – under

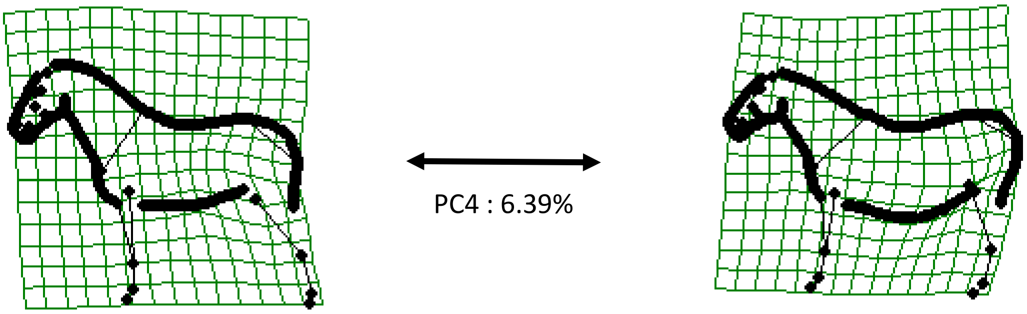

e) PC5 - Hind limb camped out – under

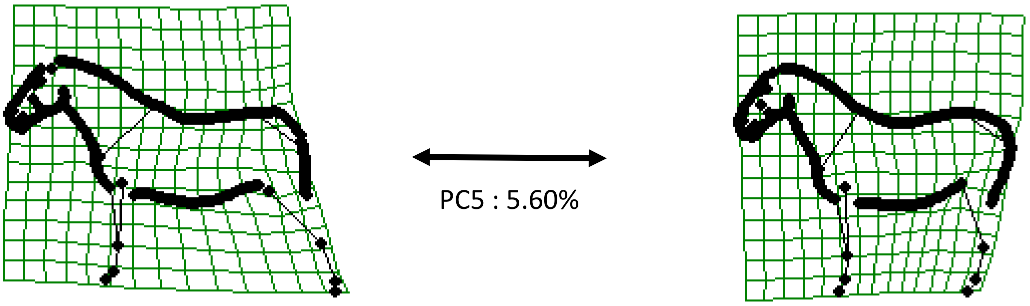

f) PC6 - Sway back to roach back

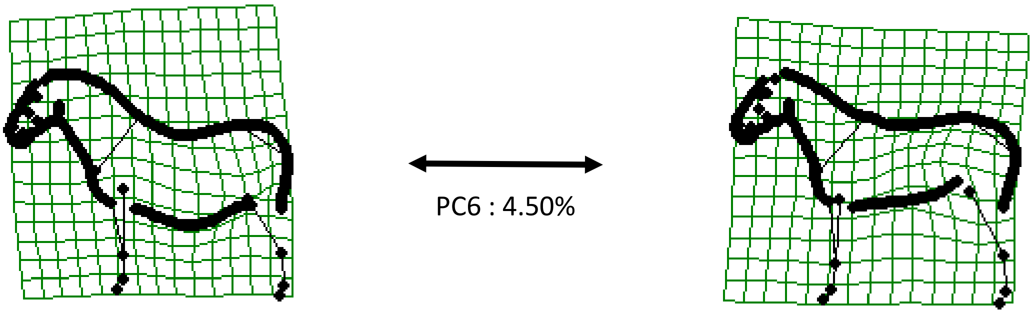

g) PC6 - « Classical » type to « Arabian » type

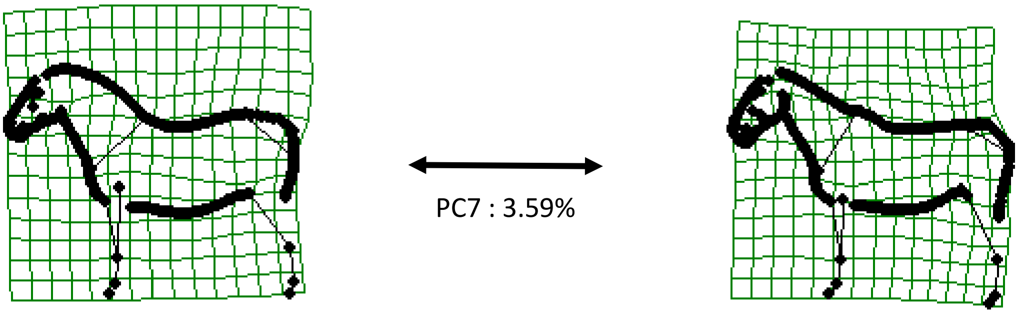

h) PC8 - front limb camped under - out

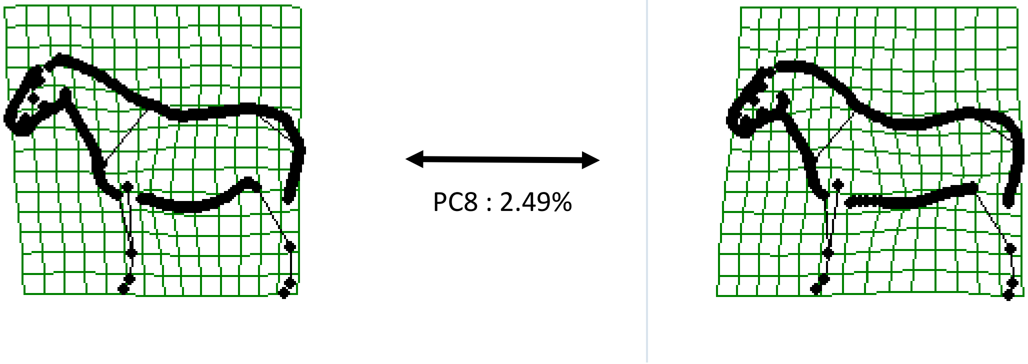

i) PC9 - slight flexion-extension at poll

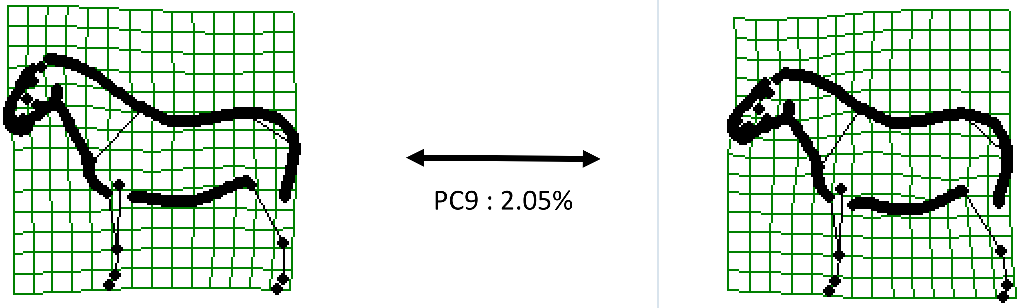

j) PC10 - Hind limb camped under – out (slight)

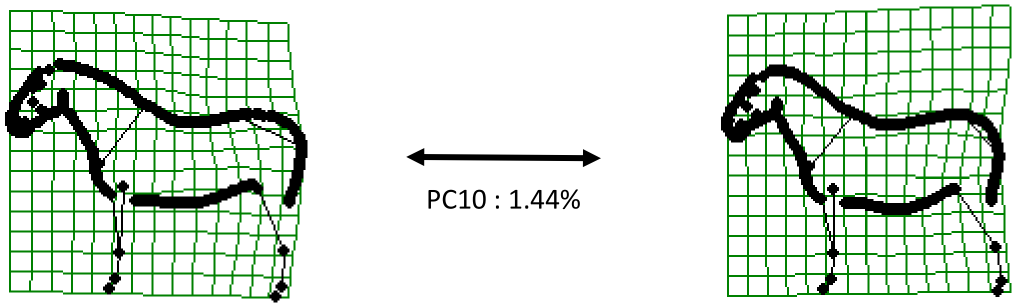

k) PC11 - Type (heavy – light)

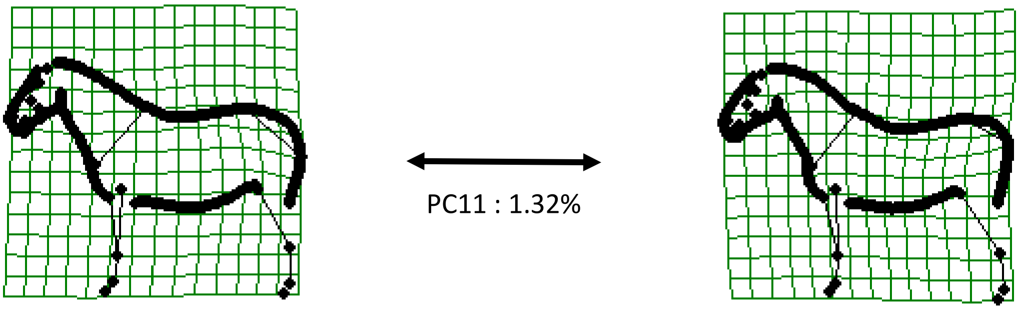

l) PC12 - Camped out – under (front and hind)

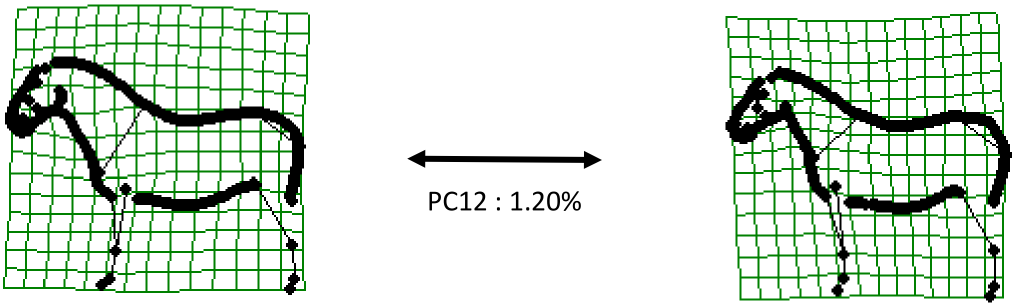

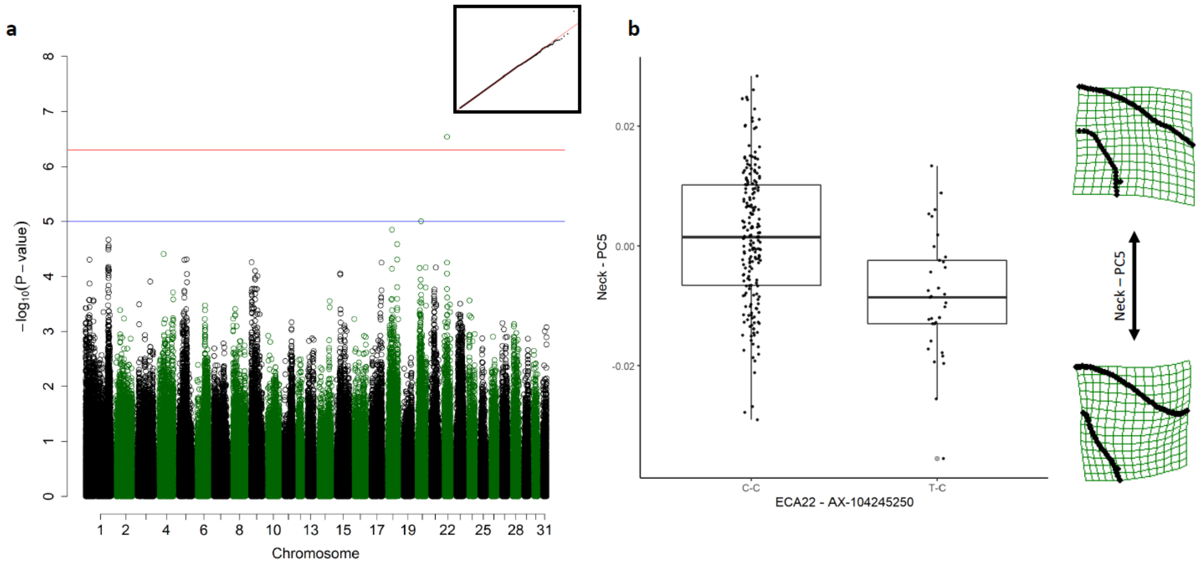

Supplementary Figure 2 : **Genome-wide association study (GWAS) for the scores from the fifth principal component of the neck landmarks (Neck PC5)**. (A) Manhattan plot with the red line representing the significance threshold corrected for the effectively independent single nucleotide polymorphisms (SNPs) ($p_{ind} < 4.98 \times 10^{-7}$). The inset on the right-hand corner shows the quantile-quantile (Q-Q) plot with the observed p-value plotted against the expected one. (B) Boxplots representing the genotype effect of the SNP on chromosome 22 on the shape of the neck. The horizontal line shows the median, the box extends from the lower to the upper quartile, and the whiskers to 1.5 x the interquartile range above the upper quartile or below the lower quartile. On the right-hand side, the extreme shapes for PC5 of the neck (shape of the neck with the highest PC5 score on top, shape of the neck with the lowest PC5 score on the bottom) are visualised in deformation grids.

Supplementary figure 3

A : mean Lipizzan horse out of 229 individuals. The horse shape is composed of 246 landmarks after generalised Procrustes analysis and sliding of semi-landmarks according to Druml et al. 2015.

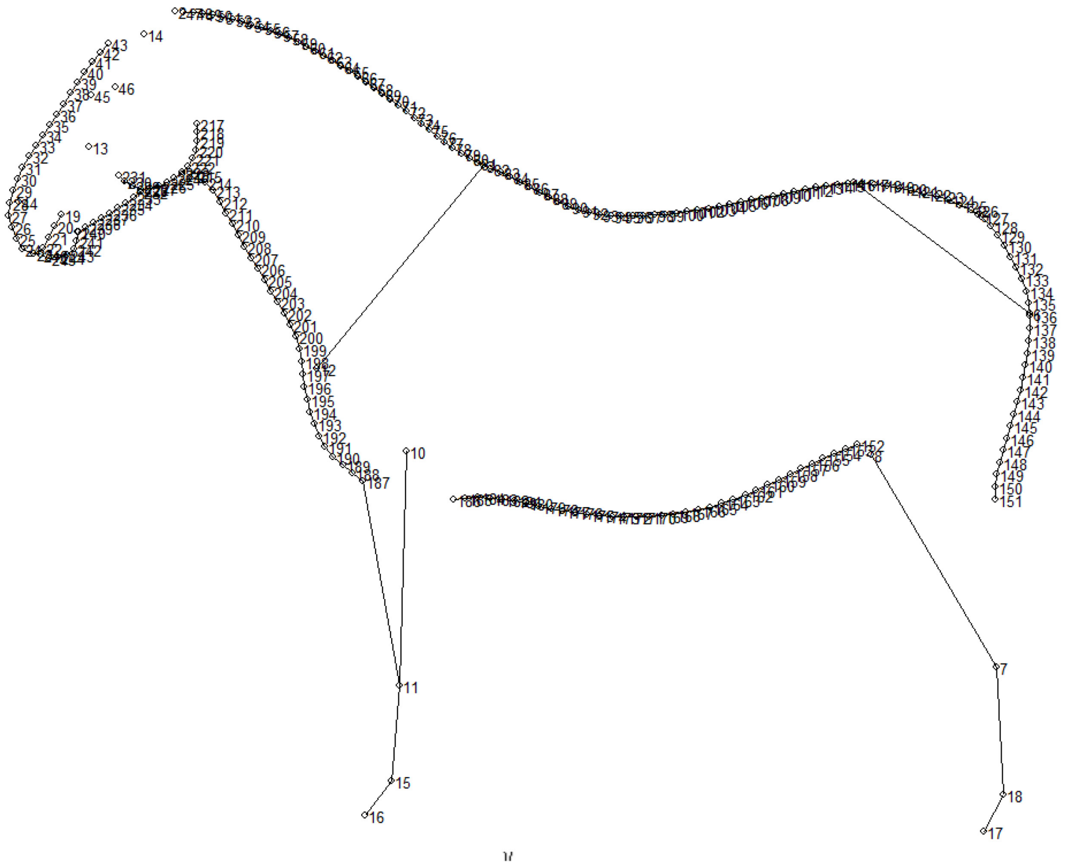

B: Subset of neck landmarks from the full horse shape space model. The landmarks selected were: 2, 3, 12, 47-91, 194-216.

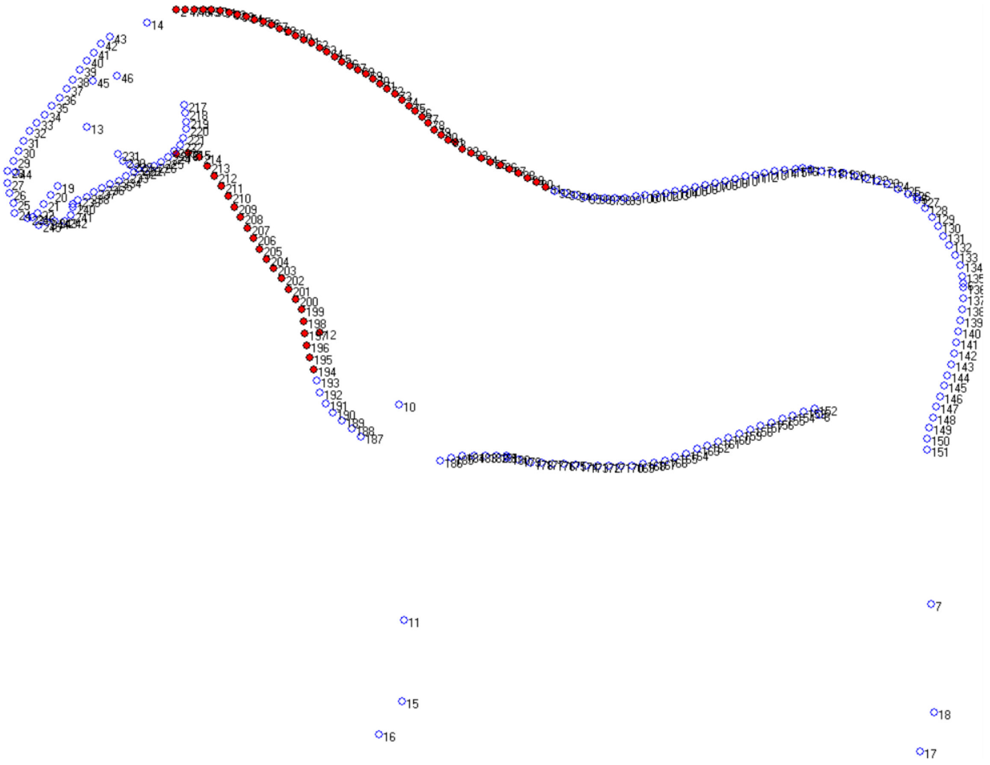

C: Subset of torso landmarks from the full horse shape space model. The landmarks selected were: 3, 4, 5, 6, 8, 9 10, 12, 80-126, 152-200.

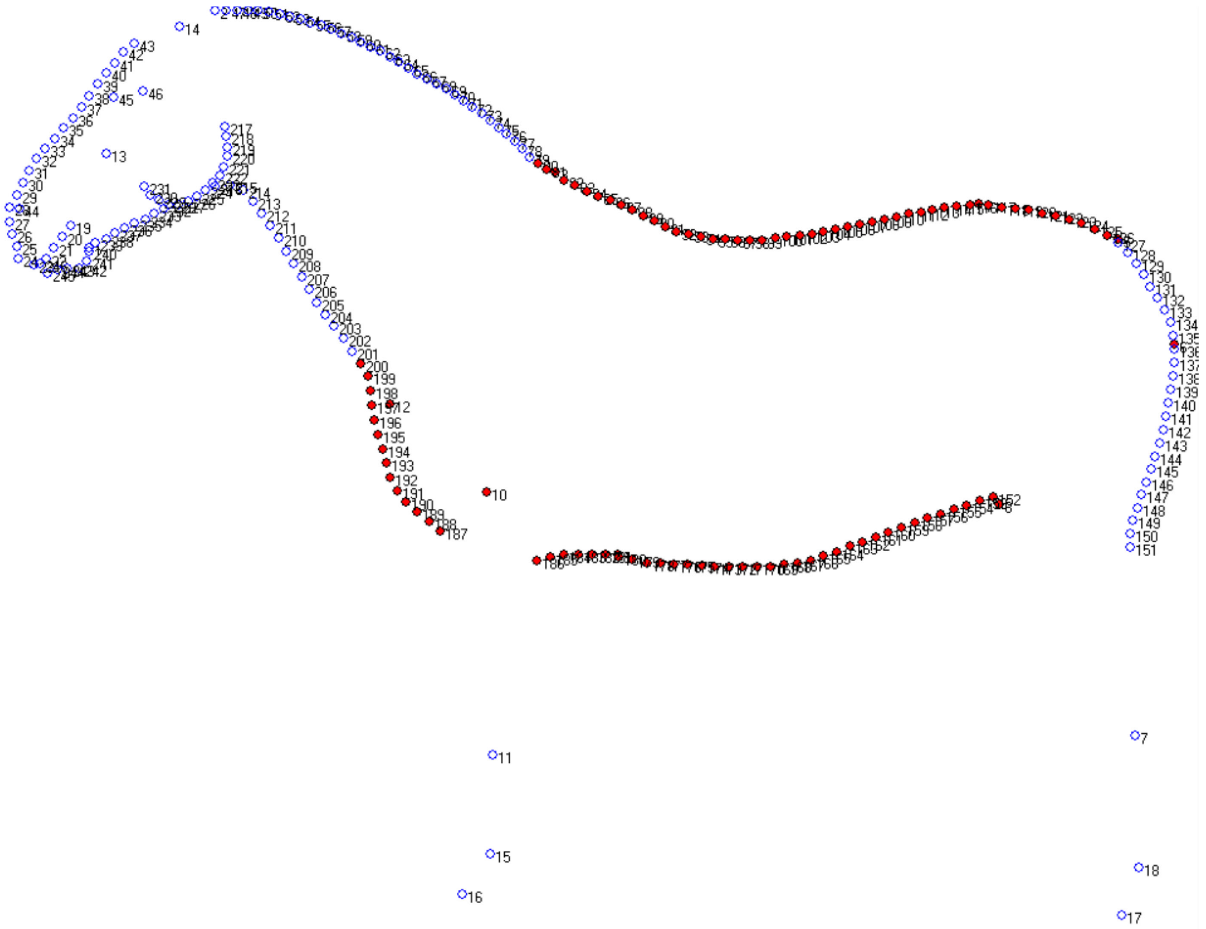

Supplement: Supplementary file 1 — Supplementary Figures. [file 41598_2023_36272_MOESM1_ESM.pdf]
